# Rebooting life: engineering non-natural nucleic acids, proteins and metabolites in microorganisms

**DOI:** 10.1186/s12934-022-01828-y

**Published:** 2022-05-28

**Authors:** Shriya Hans, Nilesh Kumar, Nisarg Gohil, Khushal Khambhati, Gargi Bhattacharjee, Shalini S. Deb, Rupesh Maurya, Vinod Kumar, Shamlan M. S. Reshamwala, Vijai Singh

**Affiliations:** 1 MTech programme in Bioprocess Technology, Institute of Chemical Technology, Mumbai, Maharashtra 400019 India; 2 DBT-ICT Centre for Energy Biosciences, Institute of Chemical Technology, Nathalal Parekh Marg, Matunga (East), Mumbai, Maharashtra 400019 India; 3 Department of Biosciences, School of Science, Indrashil University, Rajpur, Mehsana, Gujarat 382715 India; 4 Present Address: Syngene International Limited, Bangalore, Karnataka 560099 India; 5 Centre for Climate and Environmental Protection, School of Water, Energy and Environment, Cranfield University, Cranfield, MK43 0AL UK

**Keywords:** Microbial cell factories, Metabolic engineering, Non-canonical amino acids, Xeno nucleic acids, Biofuels, Non-natural biomolecules

## Abstract

The surging demand of value-added products has steered the transition of laboratory microbes to microbial cell factories (MCFs) for facilitating production of large quantities of important native and non-native biomolecules. This shift has been possible through rewiring and optimizing different biosynthetic pathways in microbes by exercising frameworks of metabolic engineering and synthetic biology principles. Advances in genome and metabolic engineering have provided a fillip to create novel biomolecules and produce non-natural molecules with multitude of applications. To this end, numerous MCFs have been developed and employed for production of non-natural nucleic acids, proteins and different metabolites to meet various therapeutic, biotechnological and industrial applications. The present review describes recent advances in production of non-natural amino acids, nucleic acids, biofuel candidates and platform chemicals.

## Introduction

**Fig. 1:**
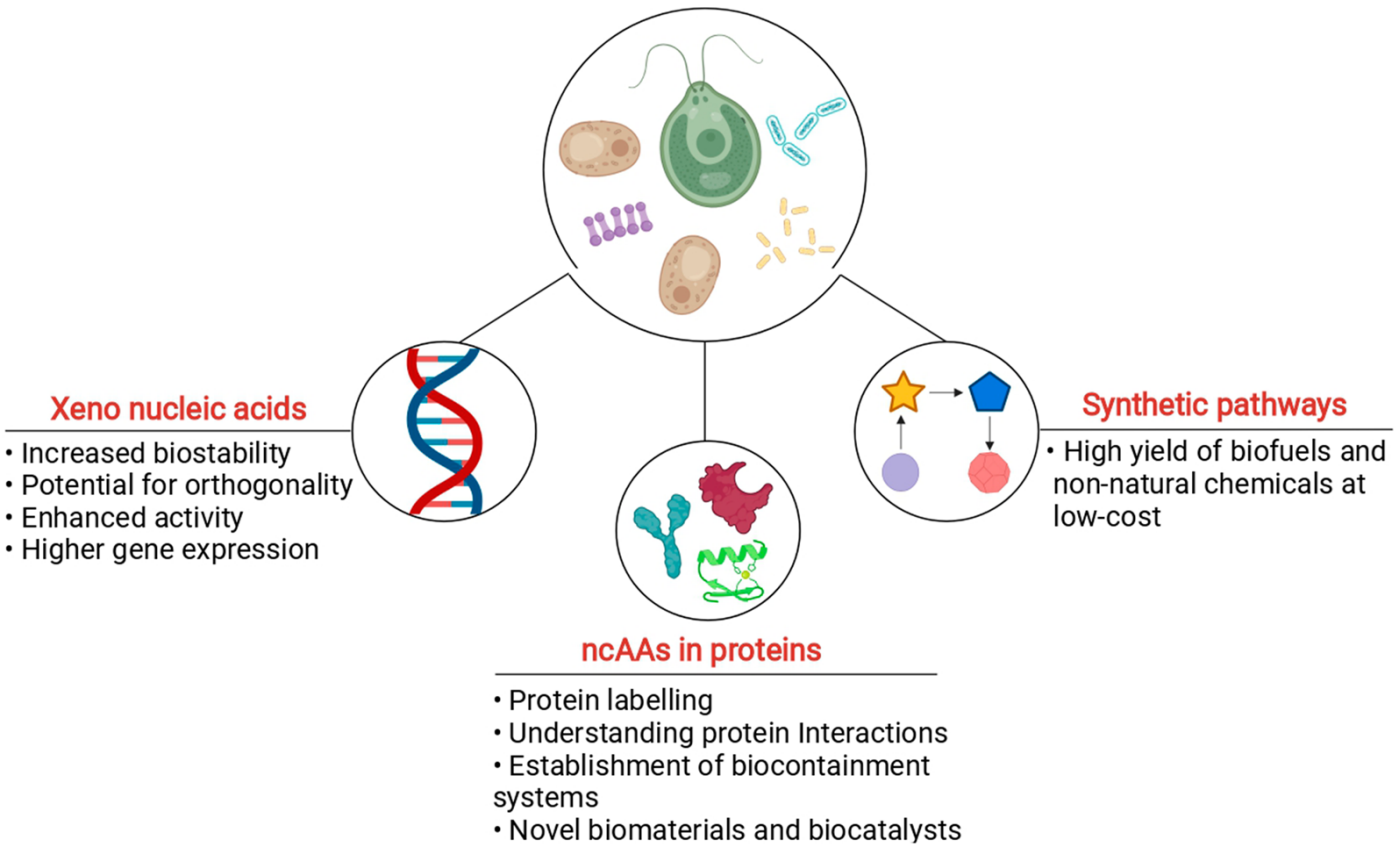
Schematic representation of utilities of non-natural entities. The figure was created with Biorender.com

The global trend towards production of natural molecules from renewable and sustainable sources has increased seen a gradual increase over the past decades. The technological advancements in metabolic engineering and synthetic biology toolboxes have opened up newer avenues for the production of natural as well as non-natural metabolites [1–3]. Non-natural metabolites exhibiting higher effectiveness could add more values in fulfilling various industrial and medicinal purposes. Given the advantages and possibilities that non-natural entities such as nucleic acids, proteins, metabolites and value-added chemicals endow (Fig. 1), the development of efficient microbial chassis that produces the same is of higher interest [1, 4].

Non-natural molecules or chemicals can be defined as those that are not found in nature but it can be synthesized using host organisms by introducing heterologous enzymatic pathways and combinatorial approach of metabolic engineering [5, 6]. Metabolic rewiring or assembling heterologous pathways in a microbial chassis is usually employed to produce non-natural molecules [7]. The primary goals of metabolic rewiring for biosynthesizing a novel or non-natural molecule include utilization of a wide range of inexpensive substrates, directing metabolic fluxes toward target products, improving stress tolerance to inhibitory products, and reducing upstream and downstream processing costs [8–11].

A combination of protein engineering, systems and synthetic biology, metabolic engineering and evolutionary engineering, along with an in-depth analysis of metabolic and regulatory networks, results in optimization of the production process by constructing efficient pathways [1, 9, 10, 12, 13]. Various strategies have been adopted to successfully implement the combinatorial approach, such as de novo pathway design, enzyme engineering, and in silico pathway prediction [10]. Computer-based prediction of synthetic pathways involves identification of possible pathways based on chemical transformation, thermodynamic favorability, enzyme docking with substrates, and the number of reactions. The most suitable organism is then selected for synthetic expression of the desired target product [1, 10]. This combinatorial approach results in the rapid development of highly-efficient microbial cell factories capable of producing a variety of value-added compounds with no known natural biochemical routes for their production.

Research towards production of non-natural metabolites has gained momentum in recent years owing to their economic relevance. *Escherichia coli* and *Saccharomyces cerevisiae* are the most widely used host for metabolic rewiring [14]. Both conventional and inexpensive substrates have been utilized in the production processes and systems biology strategies such as promiscuous enzyme screening, pathway balancing, in silico flux response analysis and thermodynamic analysis [12, 15]. For example, 1,2,4-butanetriol is a non-natural compound used in the pharmaceutical industry and is a precursor of the plasticizer 1,2,4-butanetriol trinitrate. Metabolically engineered *S. cerevisiae* has shown to improve 1,2,4-butanetriol production by expressing 2-ketoacid decarboxylase from *Lactococcus lactis* [16, 17]. Several other host microorganisms have been employed for the production of non-natural metabolites such as, the examples of which are *Corynebacterium glutamicum* and *C. crenatum* for 2-methyl-1-butanol [18, 19], *S. cerevisiae* for 1,2,4-butanetriol [20], and  *Cupriavidus necator* for 1,3-butanediol [21], etc.

The production of non-natural amino acids has also been an interesting area of research as they find many applications in therapeutics and bioplastics. 6-aminocaproic acid and 5-aminovaleric acid are two such non-natural amino acids synthesized by employing metabolic pathway engineering of *E. coli* and *C. glutamicum*, respectively [22, 23]. Genomes composed of non-natural nucleic acids are postulated to increase biostability. These are formed by incorporating non-natural base pairs by alternate hydrogen bonds, altering the phosphodiester bonds or substitution of the funarose ring structure [24, 25].

In this review, we discuss recent advances in metabolic rewiring to develop non-natural molecules. We focus our attention on the latest biotechnological developments in producing non-natural metabolites, amino acids and nucleic acids.

### Production of non-natural metabolites

Bio-based platform chemicals represent the group of molecules possessing multiple functional groups capable of producing a wide range of value-added products [26]. Bio-based non-natural platform chemicals use as biofuels, bioplastics, industrially-valuable chemicals and biopharmaceuticals [27–29]. The best-studied production hosts for non-natural chemicals are *E. coli*, *S. cerevisiae* and *C. glutamicum* [14, 30]. Table 1 lists non-natural platform chemicals and strategies used for their microbial production.

**Table 1:** Non-natural platform chemicals and strategies used for microbial rewiring

**Target product**	**Host strain**	**Metabolic rewiring strategies**	**Fermentation conditions**			**Titer (g/L)**	**References**
			**Culture**	**pH**	**Temp (**^**o**^**C)**		
1,3-BDO	*E. coli*	Addition of ADH, Nox, DERA, AKR and FDH	Batch	7.4	29	7.7	[105]
	*E. coli*	Overexpression of DERA genes	Batch (500 mL bioreactor)	7.0	37	1.1	[106]
	*C. necator*	Overexpression of *phaA* and *phaB1*; expression of *bld, dra*; deletion of *sucCD* and *phaC1*	Batch	6.9	30	2.97	[21]
1,4-BDO	*E. coli*	Overexpression of ribose 5-phosphate (R5P)-dependent PLP pathway from *B. subtilis* involving two enzymes i.e., PdxS and PdxT	Batch	7.0	37	1.41	[14]
1,5-PeDO	*E. coli*	Enhancement of downstream lysine pathway, desensitize lyine mediated feedback inhibition and deletion of *iclR*	Batch (Shake- flask)	7.0	37	0.97	[14]
	*E. coli*	Artificial metabolic modules were used to successively convert lysine into 5-hydroxyvalerate and 1,5-PeDO, overexpression of *pntAB*	Batch (Shake- flask)	7.0	37	0.39	[27]
4-Methyl-pentanol	*E. coli*	Design of high yielding pathway with enzymes selected from nine different organisms	Batch (Culture tube)	-	30	0.193	[43]
2‑Methyl‑1‑butanol	*C. crenatum*	Construction of metabolic pathway using *ILV2, ILV3, ILV5, kivd* and *adh2* genes; fermentation conditions optimization of pH, temperature, incubation time and IPTG concentration	Batch	6.5	32	4.87	[19]
Malonic acid	*E. coli*	Introduction of β-alanine pyruvate transaminase (PA0132) from *P. aeruginosa* and semialdehyde dehydrogenase; deletion of the *ydfG* gene	Fed-batch	7.0	37	3.60	[35]
6-aminocaproic acid	*E. coli*	Overexpression of L-lysine α-oxidase, series of chain elongation cycles and an aldehyde dehydrogenase	Batch	NA	37	0.046	[33]

*ADH* alcohol dehydrogenase, *Nox* NADH Oxidase, *DERA* 2-deoxy-d-ribose-5-phosphate aldolase, *AKR* aldo-ketol reductase, *FDH* formate dehydrogenase, *phaA* acetyl-CoA acetyltransferase, *phaB* acetoacetyl-2 CoA reductase, *bld* 3-hydroxybutyryl-CoA dehydrogenase, *dra* deoxyribose-5-phosphate aldolase, *pdxS* PLP synthase subunit, *pdxT* glutamine hydrolase subunit, *pntAB* transhydrogenase, *ILV2* acetolactate synthase, *ILV3* dihydroxy acid dehydratase, *ILV5* ketol-acid reductoisomerase, *kivd* alpha-ketoisovalerate decarboxylase and *adh2* alcohol dehydrogenase, *ydfG* MSA reductase

Diols and their derivatives are compounds with two hydroxyl groups and are used as chemicals, polymers and biofuels [29]. A variety of approaches have been used for the development of strains for synthesis of non-natural diols. Ethylene glycol, 1,2-propanediol, 1,3-propanediol, 2,3-butanediol, 1,3-butanediol, 1,4-butanediol, 1,3-pentanediol, 1,5- pentanediol and 2,4-pentanediol are some examples of diols, of which the first four diols are produced via natural metabolic pathways while the rest are biosynthesized by altering and modifying existing pathways or via synthetic pathways [14, 31].

Industrially important 1,3-diols have been produced by assembling synthetic pathways in several host organisms. In a study, a pathway for (*R*)-1,3-butanediol production was modified to include *bktB* (thiolase) and *phaB* (NADPH-dependent acetyl-CoA reductase) from *Ralstonia eutropha*, *pct *(propionate Co-A transferase) from *Megasphaera elsdenii*, *bed* (butyraldehyde dehydrogenase) from *Clostridium saccharoperbutylacetonium*, along with alcohol dehydrogenase from *E. coli*. This study reported the first-ever microbial production of 1,3-pentanediol and 4-methyl-1,3-pentanediol [31]. The natural metabolic pathway for the production of 1,5-pentanediol is not known. A group of researchers developed a non-natural pathway for the production of 1,5-pentanediol by utilizing low-cost carbohydrates as feedstock. Strategies comprising systematic enzyme screening, transporter engineering and pathway balancing were employed to engineer *E. coli* [27]. Wang et al*.* [14] constructed non-natural pathways having the potential of converting amino acids to C3-C5 diols. A platform was curated that led to the conversion of seven amino acids into diols such as 1,3-propanediol, 1,4-butanediol and 1,5-pentanediol from glucose.

6-aminocaproic acid is a non-natural straight chain amino acid that acts as a monomer for polymer synthesis. Turk and co-workers [32] pioneered a study on the biosynthesis of 6-aminocaproic acid via fermentation. Engineering the metabolic pathway, enzyme characterization and insersion of the expression cassettes into the microbial host led to production of 160 mg L^−1^ of 6-aminocaproic acid in laboratory-scale batch fermentations. Another group of researchers studied the artificial iterative carbon-chain extension cycle for non-natural amino acid production, wherein they used α-ketoacid as substrate for LeuABCD-catalyzed carbon-chain extensions. This study presented a novel strategy for producing non-natural straight chain amino acids from renewable feedstock using metabolic engineering [33].

Another non-natural chemical, 2,4-dihydroxybutyric acid (DHB), is a valuable precursor for the synthesis of a 2-hydroxy-4-(methylthio)butyrate methionine analogue. In a study, computer-aided engineering of template enzyme was carried out using sterically cognate substrates. Mutant libraries were generated by molecular modelling and structural analysis revealed involvement of three enzymes, i.e., malate kinase, malate semialdehyde hydrogenase and malate semialdehyde reductase. When the pathway was expressed in *E. coli,* 1.8 g L^−1^ titer of DHB was obtained [34]. Malonic acid is yet another chemical used in various industrial processes and to further produce value-added compounds. In a study, *E. coli* was engineered to produce malonic acid through the β-alanine route. Candidate semialdehyde dehydrogenases was screened for the production of malonic acid, out of which the best producing combination of enzymes was selected and introduced in a β-alanine producing strain. Fed-batch cultivation was carried out to demonstrate the production of malonic acid [35].

The several pathways, namely, isoprenoid pathway, keto-acid pathway, CoA-dependent reverse β-oxidation, fatty acid biosynthesis pathway and polyketide biosynthesis pathway have been used to produce non-natural biofuel candidates [36–38]. Low yields, high-cost and formation of undesirable side-products are major bottlenecks that can be eliminated by fine-tuning gene expression and screening the best combination of genes to yield the desired biofuel [39, 40]. In an interesting study, biosynthesis of the non-natural biofuel candidate 2-methyl-1-butanol (2-MB) was carried out in *C. crenatum* by introducing a synthetic metabolic pathway. Production of 2-MB was further enhanced by using factor combination design (FCD). The FCD consisted of four optimized parameters: pH, IPTG concentration, fermentation temperature and incubation time. It was observed that the predicted value of FCD was consistent with the higher yield of alcohols [19].

Several studies have reported the production of non-natural biofuels from the isoprenoid (terpenoids or terpenes) pathway by overexpressing endogenous genes of the deoxyxylulose-5-phosphate pathway responsible for development of isoprenoid backbone in prokaryotes, introducing the mevalonate pathway responsible for the same in eukaryotes, and optimizing non-native genes [41]. High-yield production of three C5 alcohols, 3-methyl-3-buten-1-ol, 3-methyl-2-buten-1-ol and 3-methyl-1-butanol, has been achieved by engineering the heterologous isoprenoid pathway in *E. coli*. The Shine-Dalgarno sequence of *nudB*, a phosphatase that was the main bottleneck of the pathway, was engineered. This led to a 9-fold increase in protein production. Mevalonate kinase expression was also optimized to achieve 2.23 g L^−1^ of 3-methyl-3-buten-1-ol, 150 mg L^−1^ of 3-methyl-2-buten-1-ol, and 300 mg L^−1^ of 3-methyl-1-butanol [42].

Advanced biofuel production from the keto-acid pathway starts with decarboxylation of keto-acids and further its reduction to form long-chain alcohols. A study demonstrated the production of 4-methyl-1-pentanol (4-MP) from engineered *E. coli* using the keto-acid pathway. In this study, retro-biosynthetic screening was used to design a high-yielding modular pathway for production of 4-MP as it enables exploration of enzyme diversity for greater conversion efficiency. The pathway modification involved selection of enzymes from nine different organisms to form an extended de novo pathway to produce 4-MP. The production pathways were designed and structured into four modules for identification of the most suitable enzymes to achieve high titer of 4-MP [43].

CoA-dependent reverse β-oxidation and fatty acid biosynthesis is used for the production of non-natural alcohols with the help of gene mining and engineering. A study attempted fine-tuning the specificity of *L. lactis* ketoisovalerate decarboxylase towards 1-pentanol. The enzymes encoded by *leuABCD* use acetyl-CoA to catalyze the elongation of 2-keto-acid. Saturated mutagenesis of the key residue, V461, led to a substantial increase in 1-pentanol selectivity. In situ, oleyl alcohol extraction was used to obtain a final titer of 4.3 g L^−1^ of 1-pentanol [44]. In another study, selective production of gasoline-range alkanes was achieved by engineering *E. coli*. The reverse β-oxidation synthesis route was used as an efficient alternative as compared to the fatty acid synthesis route to produce propane, butane and pentane. The  conversion of specific free fatty acids to alkanes was carried out by broad-specificity carboxylic acid reductase and mutant cyanobacterial aldehyde decarboxylases [45].

Several studies have reported modifying the intrinsic metabolic pathways of various cyanobacterial species for the photosynthetic conversion of carbon dioxide to a variety of chemicals. In one such study, synthetic metabolic pathways were introduced in *Chlamydomonas reinhardtii* and *Synechocystis* sp. PCC 6803 leading to an increase in hydrocarbon accumulation by 8- and 19-fold, respectively [46]. The first reported production of fatty acid ethyl esters (FAEEs) from carbon dioxide was obtained in a model cyanobacterium, *Synechococcus elongatus* PCC 7942. A heterologous wax ester synthase (AftA) was expressed in the cyanobacterium, wherein, introduction of ethanol production pathway in the cell lead to production of FAEEs; further enhancement in the production of FAEEs was achieved by expressing a heterologous phosphoketolase pathway [47].

### Production of non-canonical amino acids and incorporation in proteins

Non-natural (or non-canonical) amino acids (ncAAs) are incorporated into proteins to enhance or modify their properties [48]. To date, more than 200 ncAAs have been successfully incorporated in prokaryotic and eukaryotic organisms [49–52]. Incorporation of ncAAs has been explored for multiple applications, including protein labelling [53]; biomolecular targeting by binding and reacting with fluorescent probes [54–56]; identifying and understanding protein interactions [57–59]; real-time tracking and in vivo imaging [60, 61]; establishment of biocontainment systems [62, 63]; making novel biomaterials [64]; developing antimicrobial peptides [65–67]; and for generating novel biocatalysts [68–70].

**Fig. 2:**
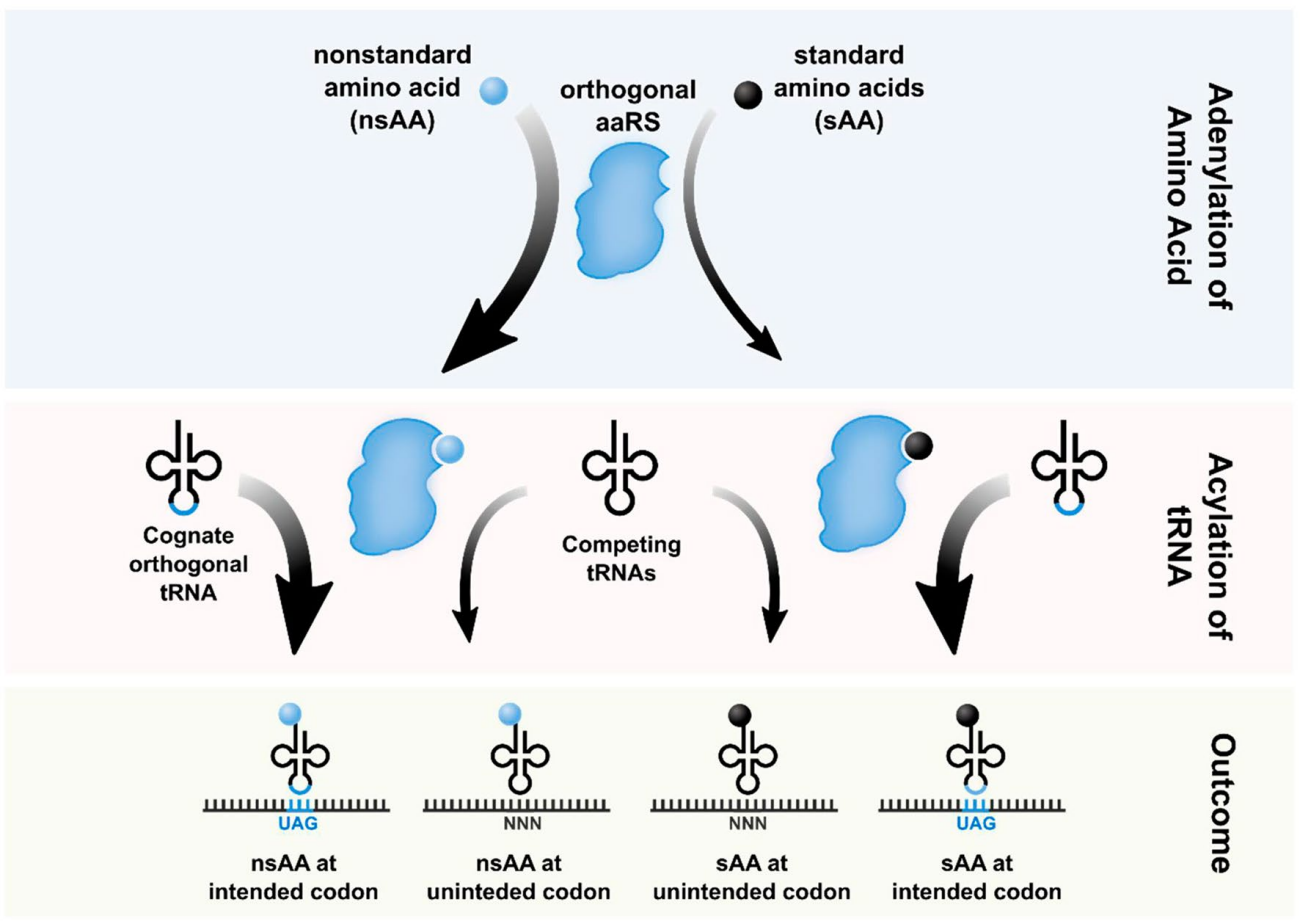
Expanding the genetic code via incorporation of nonstandard amino acids (nsAAs) into proteins. For successful site-specific insertion of ncAAs in proteins, an orthogonal tRNA/aminoacyl-tRNA synthetase (aaRS) pair is required that aminoacylate and incorporate nsAA or chemically close standard amino acid (sAA); without being recognized by the native tRNA and aaRS. The figure is adopted from Arranz-Gibert et al. [78] ^©^MDPI

For site-specific incorporation of ncAAs in proteins, an orthogonal tRNA/aminoacyl-tRNA synthetase (aaRS) pair that recognizes nonsense (usually amber stop codon (UAG)), rare or quadruple codons (e.g., UAGN, AGGA) is used [71, 72]. The non-sense codons are widely used because of their simplicity. In such system, the suppressor tRNA is used for incorporation of ncAAs by recognition of non-sense codons. However, one of the drawbacks of such system is that the ncAA would also be incorporated to the site where the non-sense codon such as amber codon terminates the protein synthesis. Rare codons are the sense codons that are rarely used in the organisms and their corresponding tRNA is present in meagre amount. For example, arginine and proline codon in *E. coli* are rarely used and therefore can be directly used for site specific incorporation of ncAAs. The same can also be used for extending the codon. For example, a stretch of mRNA sequence consisting of 4 or 5 base codons, could be recognised by extended aa-tRNA anticodons and used for site specific incorporation of ncAAs in the protein of interest. One of the advantages of using such a system is that inclusion of ncAAs and thereby the extension of the reading frame prevents the premature termination of the protein synthesis instead of the conventional halt as read by the endogenous tRNA [73]. However, orthogonal pairs need to work proficiently without being recognized by the native tRNA and aaRS [74, 75]. This requires screening of compatible orthogonal pairs that have high levels of read-through of targeted codons [74] (Fig. 2). Four major orthogonal tRNA-aaRS pairs, namely, *Methanocaldococcus janaschii* tyrosyl-tRNA synthetase (*Mj* TyrRS)-tRNA_CUA_ for *E. coli*, *E. coli* TyrRS–tRNA_CUA_ and *E. coli* leucyl-tRNA_CUA_ (*Ec* LeuRS-tRNA_CUA_) for yeast and eukaryotes, and *Methanosarcina* pyrrolysyl-tRNA_CUA_ (*M* PylRS-tRNA_CUA_) for bacteria, eukaryotes and animals, are extensively used for site-specific incorporation of ncAAs [48, 76–78].

ncAAs can be either produced within the cell or can be supplied externally in the growth medium. Several bacterial strains have been engineered for production of ncAAs [79, 80]; for example, production of L-azidohomoalanine (AHL), a methionine analogue, can be synthesized from L-homoserine by expressing the sulfhydrylation pathway in *E. coli* [81]. Elimination of methionine synthase (*metE*) enables efficient incorporation of AHL over its natural analogue into recombinantly expressed protein [81].

L-2-aminobutyric acid (L-ABA) is a ncAA that can be biosynthesised in *E. coli* overproducing L-threonine. L-ABA can be produced from L-threonine via formation of 2-ketobutyric acid by overexpression of *leuDH* from *Thiobacillus intermedius* [82]. Another ncAA, S-phenyl-L-cysteine, can be biosynthesized within the cell by manipulating the cysteine biosynthetic pathway [83].

The efficiency and specificity of aaRS can be enhanced by mutating its active site [84]. Another proven strategy is to create a library of diversified aaRS employing computational enzyme design methods, resulting in increased efficiency of insertion by up to several folds [84]. Optimization of the fermentation medium and supplementing ncAAs (1–10 mM) could also improve protein expression by many folds [85]. Some recent studies on incorporating ncAAs in proteins using different orthogonal pairs in various bacterial systems are listed in Table 2.

**Table 2:** Recent studies on expansion of the genetic code in bacteria and yeast

Host organism	Orthogonal pair	Codon	ncAA(s)	Reference
*E. coli*	Archaeal PylRS-tRNA	UAG	BocK	[65]
	*Mj*TyrRS-tRNA	AGGA	pAzF, OpgY, pAcF and pBpa	[88]
	BocLysRS-tRNA	AGGA	BocK	[72]
	AcPheRS-3-tRNA	UAGA	pAcF	[72]
	*Mj*TyrRS-tRNA/*Mb* PylRS-tRNA	UAG	pAcF, pAzF and ProcK	[67]
	Evolved *p*-cyano-L-phenylalanyl aminoacyltRNAsynthetase (*p*CNFRS)	UAG	pAzF	[108]
	Evolved *N*^*ε*^-acetyl-lysyl aminoacyl-tRNAsynthetase (AcKRS)	UAG	*m*IF	[108]
	*Mm* PylRS^TF^-tRNA	UAG	eBocK, endo-BCNK and TCO*-AK	[53]
	*M* PylRS-tRNA	UAG	BocK and AcK	[85]
	*M*ΔNPylRS/^ΔNPyl^tRNA	UAG	NmH and CbzK	[109]
	Mutant *Mm*PylRS-tRNA	UAG	PrDiAzK	[110]
*L. lactis*	Archaeal PylRS-tRNA	UAG	BocK	[65]
*Streptomyces albus*	*Mb* PylRS-tRNA	UAG	Alk, Cyc and Boc	[51]
*S. elongatus*	*Mm* PylRS-tRNA	UAG	PrLl and BocK	[104]
*Neisseria meningitides*	*M* PylRS-tRNA	UAG	mAzZLys and pBpa	[52]

*BocK* *N*ε-(tert-butyloxy-carbonyl)-L-lysine, *pAzF* *p*-azidophenylalanine, *OpgY* *O*-propargyl-tyrosine, *pAcF* *p*-acetylphenylalanine, *pBpa* *p*-benzoyl-phenylalanine, *ProcK* *N*ε-prop-2-ynyloxycarbonyl-L-lysine, *mIF* *m*-iodo-L-phenylalanine, *eBocK* tetrazine-unreactive eBoc-lysine, *endo-BCNK* tetrazine reactive endo-bicyclo [6.1.0] nonyne-lysine, TCO*-AK axial isomer of trans-cyclooct-2-ene-lysine, *AcK* *N*ε-acetyl-L-lysine, *NmH* 3-methyl-L-histidine, *CbzK* *N*ε-(carbobenzyloxy)-l-lysine, *PrDiAzK* propargyl-diazirine-lysine, *Alk* H-Lys-Alloc-OH, *Cyc* H-Lys-Cyc-OH, *Boc* H-Lys-Boc-OH, *PrLl* *N*ε-propargyl-L-lysine, *mAzZLys* *N*ε-(m-azidobenzyloxycarbonyl)-L-lysine

To achieve 100% efficiency of ncAA incorporation in proteins using UAG as codon, the elimination of release factor-1 (RF-1) is necessary. RF-1 recognizes UAG codon and terminates translation [86, 87]. Knocking-out RF-1 eliminates the competition between charged amber suppressor tRNA and RF-1, leading to complete re-assignment of UAG as a sense codon. However, the yield of the recombinant protein obtained is compromised as eliminating RF-1 is lethal to the cell [86]. To ameliorate this situation, 95 UAG codons in the *E. coli* genome were replaced with the other stop codons allowing safer elimination of RF-1 factor and facilitating modified protein production at larger scale [87].

The rare promiscuous activity of *Mj* TyrRS towards tRNA_UCCU_ is exploited for inserting ncAA via AGGA quadruplet codons [88]. However, arginine aminoacylates tRNA_UCCU_ and thus competes with ncAA for charging and incorporation into protein at AGGA codons. Replacing A38 base with cytosine in tRNA_UCCU_ results in decreased arginine charging and hence increased incorporation of ncAA into protein. Furthermore, the first three bases of AGGA quadruplet codons is a rare codon and can be recognized via arginine tRNA_CCU_. Knocking out arginine tRNA_CCU_ aids in efficient incorporation of ncAA via A38C tRNA_UCCU_ without affecting growth of *E. coli* [88].

### Incorporation of non-natural bases in nucleic acids

A set of new properties such as greater stability or activity can be achieved by introducing additional functional groups into DNA and RNA, the natural nucleic acids [89]. Xeno nucleic acids (XNAs) have the potential of replacing DNA and RNA to introduce desired features into the existing genome. An advantage of non-natural nucleic acids is their ability to resist degradation; therefore, expression level of inserted genes is high [90–92]. However, challenges such as biocompatibility with polymerases for recognition and efficiently converting information in the central dogma still persist and demand to be addressed [93].

**Fig. 3:**
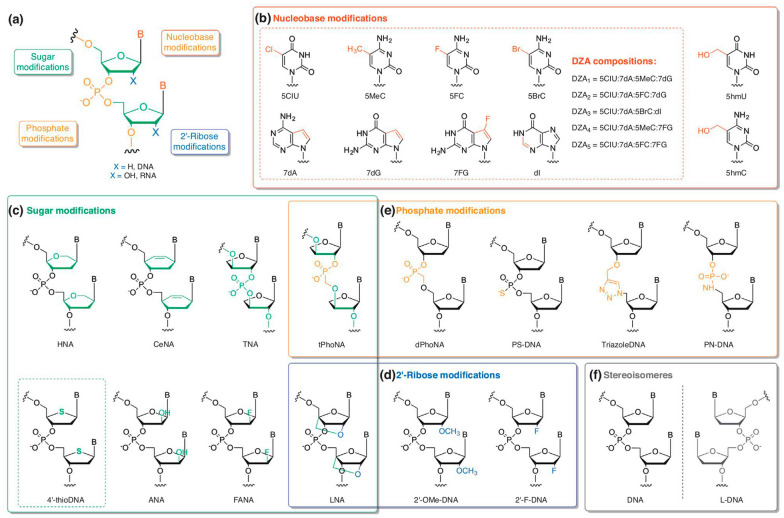
Various XNA chemical structures. **a** Different strategies incorporating possible chemical alteration in nucleic acid structure which includes nucleobase, sugar, 2ʹ-ribose and phosphate modifications **b** Examples of nucleobase modified XNA molecules, including g 5-chloro-20-deoxyuridine (5ClU), 5-methyl-20-deoxycytidine (5MeC), 5-fluoro-20-deoxycytidine (5FC), 7-deaza-20-deoxyadenosine (7dA), 7-deaza-20-deoxyguanosine (7dG), 7-fluoro-7-deaza-20-deoxyguanosine (7FG), and 20-deoxyinosine (dI). **c** Examples of sugar modified XNA molecules, including hexitol nucleic acid (HNA), cyclohexenyl nucleic acid (CeNA), threose nucleic acid (TNA), 30–20 phosphonomethyl-threosyl nucleic acids (tPhoNA). 4ʹ-ribose-modified 4ʹ-thioDNA, arabino nucleic acid (ANA), 20-fluoro-arabino nucleic acid (FANA), and locked nucleic acid (LNA). **d** Examples of 2ʹ-ribose modified XNA molecules, including LNA, 20-O-methyl DNA (20-OMe-DNA), and 20-fluoro DNA (20-F-DNA). **e** Examples of phosphate modified XNA molecules, including tPhoNA, 50–30 phosphonomethyl-deoxyribosyl nucleic acids (dPhoNA), phosphorothioate modified DNA (PS-DNA), TriazoleDNA, and PN-DNA. **f** Examples of stereoisomeres molecules, including mirror-image nucleic acid (_L_-DNA). The figure is reproduced with permission from Eremeeva and Herdewijn [24] ^©^Elsevier

Nucleic acids could be either modified by altering the nucleotide base, sugar moiety or the phosphate group (Fig. 3a) [24]. Substitution of the nucleobase can modify the base-paring properties in nucleic acids. The bases A, T, G and C are substituted or modified in the nucleotide structure. Modified bases include N6-methyl adenine, 5-methyl cytosine, 5-hydroxymethyl cytosine (Fig. 3b), 5-formyl cytosine, 5-carboxycytosine and 5-(hydroxymethyl)uridine (Fig. 3b) [94, 95]. Two studies were conducted on replacing the canonical nucleoside with the modified ones by rewiring the pyrimidine biosynthetic pathway of *E. coli* using codon-optimized bacteriophage genes. In one such study, 75% of thymidine in the genomic DNA of *E. coli* was successfully replaced with 5-(hydroxymethyl) uridine (5hmU) (Fig. 3b) [95], while in the other study, 63% of genomic 2′-deoxycytidine in *E. coli* was replaced with 5-(hydroxymethyl) cytidine (5hmC) (Fig. 3b); whereas, the same in case of plasmid happened to be 71%. Besides, the said study also reported to modify 20 and 45% of 5hmC to glucosyl-5-hydroxymethyl-2′-deoxycytidine (5-gmC) in the genome and plasmids of *E. coli,* respectively, by engineering its glucose metabolic pathway [94]. Furthermore, a random mutagenesis approach exhibited to enhance their incorporation into the genomic DNA. Modified nucleosides were found to play a vital role in regulating transcription, and stabilizing epigenetic modifications and restriction-modification systems in both prokaryotic and eukaryotic systems [94, 95].

Unnatural base pairing (UBPs) focuses on introducing a third-base pair apart from A-T and G-C into DNA. It has been demonstrated in vitro that UBPs are biologically functional during translation, transcription and replication [96]. A study demonstrated in vivo transcription of DNA in a semi-synthetic organism with the unnatural base pair dNaM-dTPT3 leading to site-specific integration of natural or unnatural amino acids in green fluorescent protein. It was observed that the semi-synthetic organism could grow robustly and stably maintain the modified genetic material [97].

XNA aptamers with sugar modifications have greater stability as well as higher affinity to target molecules. Amplification of a highly modified DNA using only unnatural dNTPs carried out by employing different DNA polymerases to incorporate 2′-deoxy-4′-thionucleoside 5-triphosphates (dSNTPs) have shown that the B DNA polymerase family is able to integrate dSNTPs by a single nucleotide insertion and primer extension. The oxygen atom in the furanose ring in 2′-deoxy-4′-thioribonucleic acid (4′-thioDNA) (Fig. 3c) is substituted by a sulphur atom. This modification in the nucleic acid structure confers higher nuclease resistance [98].

In another study, hexitol nucleic acid (Fig. 3c) (HNA)-DNA mosaic backbone was modified by incorporating 5-methyl-isocytosine and isoguanine nucleosides. The results demonstrated that the backbone scaffold of the base resulted in vivo mispairing and misincorporation, hence leading to orthogonality [99]. Other examples of non-natural nucleic acids include cyclohexenyl nucleic acid (CeNA) (Fig. 3c) [100] and 20-fluoro-arabino nucleic acid (FANA) (Fig. 3c) [101]. A novel enzyme 3′–2′ phosphonomethyl-threosyl nucleic acid (tPhoNA) (Fig. 3c and e) synthase was engineered with an already described XNA reverse transcriptase. The results demonstrated tPhoNA to be a viable genetic material [92]. Other such XNA chemistries are shown in Fig. 3 along with their structures.

In phosphate modified XNAs, the conventional phosphate diester bonds are substituted by a different functional group. A study demonstrated the extension of 3-amino terminated primers in *Bacillus stearothermophilus* by DNA polymerase. The cofactor Mg^2+^ was replaced by Ca^2+^ which led to an increase in the reaction rate by five times and the incorporation of 3′-amino-2′,3′-deoxynucleosides 5-triphosphate to yield N3′-N5′ phosphoramidate (NP) bonds. The reaction rate was observed to further increase by 21-fold by a single active site mutation. The template-directed activity led to NP-DNA backbone linkage [102]. In an interesting study, enzyme-free oligonucleotide assembly into a gene by click-DNA ligation method was described for overcoming the drawbacks of chemical synthesis of oligonucleosides. Increased biocompatibility of a triazole-containing DNA in *E. coli* was observed by making an epigenetic variant of the *iLOV* gene [103].

Artificially expanded genetic information systems (AEGIS) have been incorporated in living cells to help replicate plasmids made from such systems. This is one of the methods to expand the genetic alphabet through unnatural metabolites and systems [104]. Researchers have developed methods, applicable to almost all unnatural systems that can be used for phosphorylation of unnatural nucleotides using kinases. Synthesis and evaluation of both natural as well as non-natural deoxyribonucleoside triphosphates as polymerase substrates were carried out. 6-amino-5-nitro-3-(1′-β-D-2′-deoxyribofuranosyl)-2(1H)-pyridone and 2-amino-8-(1′-β-D-2′-deoxyribofuranosyl)-imidazo[1,2-a]-1,3,5-triazin-4(8H)-one are the two AEGIS nucleoside diphosphates. In vitro formation of dNTPs and its incorporation into DNA has already been demonstrated [105].

Non-natural nucleic acids have a number of advantages over their natural counterparts such as biostability and potential for orthogonality. Modifications in the sugar moiety, nucleobase or phosphate group of DNA lead to increased activity and stability and for the most part are recognized by the cellular machinery. UBPs are used for the development of semi-synthetic microorganisms, however, only a few unnatural nucleic acids have been reported to completely replace DNA or RNA.

### Conclusion and perspectives

This review presents recent studies on the production of non-natural molecules by metabolic engineering of host microorganisms. Incorporation of non-natural nucleic acids and ncAAs has enabled researchers to explore possibilities of novel information storage molecules and biocatalysts that can confer useful properties to cells. However, more stringent selection platforms are needed that could enhance the incorporation of non-natural nucleic acids into the host organisms without the possibility of eruption of undesirable responses. In order to achieve the same, investigation related to the response of physiological regulators which limits the replacement of natural to the non-natural moiety in the genomic template of host organisms must be implemented [95]. In addition, in an effort to replace the natural moiety with its non-natural counterpart at genomic scale, several mutations have been observed in the host organisms and thus the reduction of the same could be the future direction of work in the XNA field [93]. Apart from that, FANA has been an invaluable tool for therapeutic lead discovery, functional genomics and structural biology. However, given the fact that FANA can stabilize and tune several nucleic structures, the same has also the potential in FANA-based sensors for in-cell experiments [101]. Several strategies have also been developed for incorporation of ncAA into the proteins; however, there are few of the limiting factors that need to be addressed to achieve the same with ease and in an efficient manner. For example, the incorporation of bulky or extremely charged ncAAs into the protein is a challenging task as it is difficult for such a structure to cross the cell membrane. Propeptide strategy or engineering the host transport system for better uptake of such ncAAs could be a possible solution to incorporate them into the protein. Another limitation is the flexibility of the sequence to incorporate a mutation without significant loss of protein function. For instance, in the scenario where the stop codons are used to incorporate ncAA, it has been realised that the efficiency to achieve the same depends on the position of the mutant site as well as the nature of the protein. Multiple incorporation of ncAAs in peptide or protein of interest is also a challenging task, as for each ncAA, a unique codon must be assigned. So far, the application of frameshift suppression technique could be the possible solution for such a problem. Likewise, even though there is a repertoire of ncAAs, it appears that the entities in the same are not diverse enough. The usage of evolution techniques on orthogonal translation systems could assist to increase the repertoire and diversity of ncAAs [73]. Altogether, considering the advances gained in incorporation of ncAAs, it appears that in near future, the upcoming difficulties and the mentioned hurdles could easily be overcome. Non-natural biochemicals find their use as industrially important chemicals and have a wide applicability in biotherapeutics. The main bottleneck in developing a pathway for the synthesis of non-natural platform chemicals is either the presence of promiscuous enzymes or selection and introduction of a suitable enzyme into the pathway. Furthermore, development of more accurate genome scale metabolic models, along with the incorporation of the regulation constraint could assist to enhance the flux towards the non-natural metabolic pathways. With advances in de novo enzyme designing coupled with high-throughput screening strategies, the workflow of constructing robust host strains for production of non-natural molecules is getting streamlined and efficient. Commercial success stories which are currently few and far in between will provide the much-needed fillip for bio-based chemical production.

## Data Availability

The datasets supporting the review are included within the article.
